# BronchoX: bronchoscopy exploration software for biopsy intervention planning

**DOI:** 10.1049/htl.2018.5074

**Published:** 2018-09-18

**Authors:** Esmitt Ramírez, Carles Sánchez, Agnés Borràs, Marta Diez-Ferrer, Antoni Rosell, Debora Gil

**Affiliations:** 1 Computer Vision Center, Autonomous University of Barcelona, Bellaterra 08193, Spain; 2 Bellvitge University Hospital, L'Hospitalet de Llobregat, Barcelona 08907, Spain

**Keywords:** medical image processing, computerised tomography, pneumodynamics, medical computing, lung, path planning, image segmentation, BronchoX, navigation, PLs, biopsy intervention planning, virtual bronchoscopy, noninvasive exploration tool, VB software, medical software developers, pulmonary lesions, bronchoscopy exploration software, virtual paths planning, trachea, physician readable instructions, segmentation, visualisation, respiratory tract, bronchioles

## Abstract

Virtual bronchoscopy (VB) is a non-invasive exploration tool for intervention planning and navigation of possible pulmonary lesions (PLs). A VB software involves the location of a PL and the calculation of a route, starting from the trachea, to reach it. The selection of a VB software might be a complex process, and there is no consensus in the community of medical software developers in which is the best-suited system to use or framework to choose. The authors present Bronchoscopy Exploration (BronchoX), a VB software to plan biopsy interventions that generate physician-readable instructions to reach the PLs. The authors’ solution is open source, multiplatform, and extensible for future functionalities, designed by their multidisciplinary research and development group. BronchoX is a compound of different algorithms for segmentation, visualisation, and navigation of the respiratory tract. Performed results are a focus on the test the effectiveness of their proposal as an exploration software, also to measure its accuracy as a guiding system to reach PLs. Then, 40 different virtual planning paths were created to guide physicians until distal bronchioles. These results provide a functional software for BronchoX and demonstrate how following simple instructions is possible to reach distal lesions from the trachea.

## 1 Introduction

Bronchoscopy is an endoscopic technique that allows visualising the inside of lung airways by inserting an instrument with a camera (i.e. bronchoscope) through the nose or mouth. Bronchoscopy examinations allow biopsy of pulmonary nodules with minimum risk for patients. The main constraint of flexible bronchoscopy is the difficulty to determine the best pathway to reach peripheral lesions.

Virtual bronchoscopy (VB) [1] is a non-invasive imaging-based procedure to examine airways, and it is used to plan biopsy interventions. VB inspects lungs using a computed tomography (CT) volume of the patient, and it allows planning the procedure to be followed during the intervention. Biopsy planning involves both the location of the peripheral pulmonary lesion (PL) and the identification of the path across airways to reach it. This path would be the best suited to perform the medical procedure using the available instrumentation. In the surgery room, the intention is to follow the planning previously carried out to locate the PL, preferably following friendly instructions. This process can be supported by computer-assisted software, which is a useful tool to help physicians.

Currently, there are some commercial options for VB planning and visualisation such as Archimedes planner/system and LungPoint planner/virtual bronchoscopy navigation, both developed by Bronchus (http://www.broncus.com), or Spin System, developed by Medical Veran Technologies (http://www.veranmedical.com). The major drawbacks of adopting these systems are their set-up and establishment process into clinical environments, and their expensive licences. Therefore, exploring the available open-source medical software to be used as VB planner would be very useful. The development process of an assistance software for bronchoscopists requires the study of several options, to choose the proper medical framework that best fits the clinical requirements. However, there is not a consensus about which software should be used for a VB since it involves diverse tasks that include matching of planning with intervention videos, nodule segmentation, and location, two-dimensional (2D)/3D visualisation, and others.

This kind of software might be developed using an existing framework by adding new functionalities (as modules or extensions) or not. Undoubtedly, using a framework involves considering the steep learning curve to develop applications, the portability for different platforms, the available documentation, and the extensibility to develop new features over it. These aspects may create doubts in which is the adequate framework for a particular need or how to implement a particular algorithm in a chosen framework.

Bearing this in mind, in this Letter, we present an open source, multiplatform (Windows, Mac, and Linux) planning software for VB. The planning software allows the visualisation and navigation of pulmonary airways. It performs the segmentation of the airways and its codification as a binary tree. Moreover, it allows building a path until a PL, starting from the trachea, through the segmented airways following an airways centreline. This path is built using a virtual fly-through camera, projecting images at all bifurcation points. This image-based approach allows the generation of instructions to be used during bronchoscopy as a roadmap.

## 2 Medical imaging frameworks

There are plenty of image processing and visualisation frameworks to develop medical applications. In this research, we only focus on the open-source medical imaging libraries, application programming interfaces (APIs), or frameworks extendable by plugins or modules. In general, a medical imaging software intends should be used by a multidisciplinary team, integrated by physicians and scientists. Remarkable examples are SCIRun [2], Medical Imaging Interaction Toolkit (MITK – http://mitk.org), VolView (www.kitware.com/volview), MATLAB (https://mathworks.com), and MeVisLab (www.mevislab.de). These have been used as the basis for several developments in the medical imaging field.

Similarly, there are medical imaging packages that cover tasks such as image processing and analysis (ITK – www.itk.org), visualisation (VTK – www.vtk.org), tracking, and related tasks in image-guided surgery (Image-Guided Surgery Toolkit [3]), real-time image, and video processing (Open Source Computer Vision Library (OpenCV) – https://opencv.org), or real-time simulations (Simulation Open Framework Architecture – www.sofa-framework.org). These packages work on different levels having their strengths and weaknesses, being used for general or specific purposes in a particular task. Further studies of image processing tools for the medical application can be found in [4, 5].

Other notable software platforms for medical imaging are 3D Slicer [6] and OsiriX [7]. 3D Slicer is an open-source software for medical imaging and 3D visualisation built over two decades ago. Osirix is a suite of medical image processing and visualisation software, supporting the Digital Imaging and Communications in Medicine (DICOM) standard. Both are widely used in the medical community offering an easy integration with other hardware/software modules. They allow developing more complex and personalised applications.

Higgins [8] argues that VB applications should offer the navigation and visualisation of pulmonary structures. Also, these applications might be valuable as supporting tools for physicians, and applications would offer improvements for the clinical practise: positional information of lesions, projections, and different views of structures, and is capable to extract data to be analysed later.

The considerations suggested by Higgins represent a foundation for some studies in the development of VB solutions. In 2017, Nardelli *et al.* [9] use an architecture integrating different libraries/APIs: an electromagnetic tracking system handled with MATLAB, and a video grabber connected to 3D Slicer using Public Software Library for Ultrasound Imaging [10]; both components joined with the OpenIGTLink (http://openigtlink.org) library. Also, Python and C++ programming language were used to write its procedures and ITK for the centreline extraction. Another example is the usage of the OsiriX software, where Fiorelli *et al.* [11] present a VB tool to improve the accuracy of trans-bronchial needle aspiration for mediastinal staging, running in a tablet device.

Alternatively, some VB solutions use platforms built from scratch to ensure an optimal fulfilling of their requirements. Namely, CustusX [12] is a navigation system for image-guided intervention developed by the Norwegian National Competence Centre for Ultrasound and Image-Guided Therapy (http://usigt.org). Since 2003, CustusX has been used by clinical and technological researchers to medical imaging and navigation. It could be used as a navigation system or as a toolkit to develop new applications. In particular, Jens *et al.* present a visualisation method for navigated bronchoscopy with CustusX [13] including a technique called Anchored to Centerline Curved Surface using a curved 3D surface from the trachea through the smaller airways.

Although using medical frameworks are powerful choices to start in the development of prototypes to *in vivo* surgical applications, they are complex and often difficult to learn. In fact, Johnson [14] reported that frameworks require a good documentation and longer training than other options. Also, frameworks might be hard to develop, requiring better programmers than regular application developers.

Accordingly, the development of a VB functionality without using a framework is an option to consider. Numerous attempts have been made to develop algorithms for registration, segmentation, and planning [15–17], mostly using C++ as the core programming language and OpenGL for the visualisation. Nevertheless, one of the main drawbacks to adopt these systems is the lack of information on how to use them. In 2007, an image-guided bronchoscopy for all planning stages was developed [18], and it was used during surgery to improve the procedure success rate. However, this solution is not shown as reproducible. Similarly, other interesting proposals [19, 20] do not report the details of their implementations.

A few programming languages such as C or C++, offer performance and speed and they can nearly interface with other languages. Nevertheless, the development of medical applications using this class of language might involve a large developing time. Then, developing a short-time software prototype, without sacrificing the performance, emerges as an excellent option to develop.

The next section presents our proposal to create a planning software for bronchoscopy, using particular algorithms developed for our multidisciplinary group; covering tasks such as lung lesion selection, visualisation, airway segmentation, and simple guiding instructions for physicians. Section 4 shows our results and discussion. Finally, Section 5 concludes the Letter and future work of our research.

## 3 Bronchoscopy planning software

**Bronco**scopy E**x**ploration (BronchoX) is our proposal for a biopsy intervention planning software, with the intention to be used prior to the bronchoscopy. BronchoX integrates different aspects to consider previous surgical intervention, being a multiplatform and computer-efficient-aided tool.

The software allows loading, visualising, and processing CT volumes in DICOM format. Pulmonary airways are segmented, and they are encoded in a 3D geometry using a tree-based structure (see Section 3.2). In addition, BronchoX allows physicians to navigate interactively for the CT anatomical planes (axial, coronal, and sagittal), and select the PL (interest point). Once a target point is selected, BronchoX constructs a path to the closest bronchi or bronchiole (see Section 3.3), and generates a set of human-readable instructions to be used during the intervention (more details in Section 3.4).

**Fig. 1:**
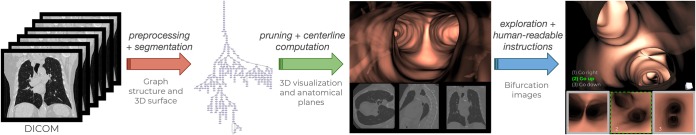
Pipeline of the VB planning system. Using patient DICOM data, a graph structure is computed from a segmentation of airways. Graph allows the encoding of any path across airways as a list of instructions at each branching point. Path instructions are visually rendered as a mosaic of the whole route for instruction guiding

Fig. 1 summarises the pipeline of BronchoX. First, the user selects the corresponding study for a patient in DICOM format (CT volume). Next, the pre-processing and airway segmentation algorithms are executed generating a 3D geometrical mesh of airways. This mesh is encoded into a rooted binary tree which represents the airways bronchi geometry set by the structure of the segmentation centreline. Thus, the visualisation of the 3D geometry and 2D anatomical slices are available. Finally, the instructions based visual roadmap from the trachea to the PL is showed to the user and exported for being used during an intervention.

### 3.1 Architecture

The layer-based architecture uses a set of well-known stable libraries in order to have a simple and reproducible software pipeline, to develop algorithms for VB planning and guiding. The design of BronchoX is based on the C++ programming language, which is used for its efficiency to handle computational resources.

We opted for QT to develop the graphical user interface (GUI), which will be directly used by physicians. This selection does not entail implications in the object-oriented programming paradigm. The GUI is fluid and intuitive for physicians offering icons as visual elements. Also, QT contains cross-platform components to create compliant software ensuring patient safety.

**Fig. 2:**
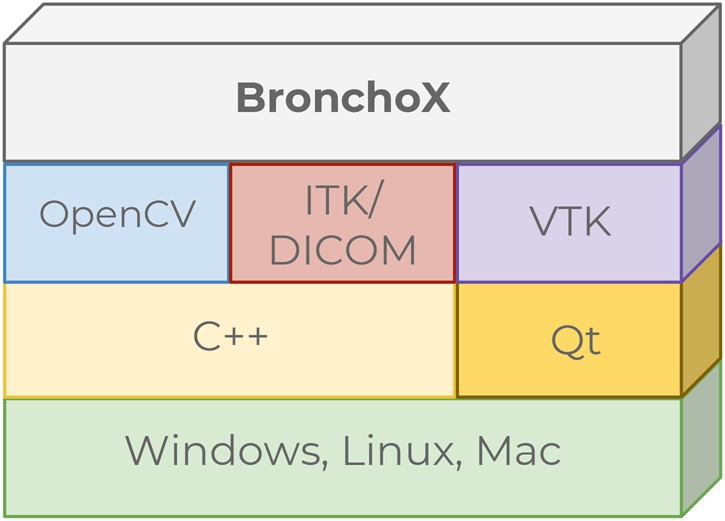
Layer-based architecture proposed in BronchoX

Fig. 2 shows the architecture where C++ programming language and QT framework are above the host operating system. The libraries OpenCV, ITK, and VTK are on the top of BronchoX implementation performing the rendering and processing of the 3D objects.

The proposed architecture allows BronchoX to function whether as a high-level library for developers or as a programming language directly. In fact, it is possible to incorporate algorithms or libraries with the C++ integration capability (e.g. MATLAB or Python scripts). The combination of algorithms offered by VTK, ITK, and OpenCV are favourable to implement particular functionalities for bronchoscopy: 3D reconstruction, DICOM handling, image filtering, and others. Furthermore, OpenCV provides modern features such as augmented reality and deep learning.

### 3.2 Anatomy segmentation and representation

Our segmentation strategy is based on the thresholding of bronchi local appearance maps computed convolving CT volumes with own-designed tubular filters, which are defined using second derivatives of Gaussian kernels. To account for the difference in calibre and appearance between main and distal airways, we adopt a multiresolution approach.

The segmented anatomy is encoded as considering any possible path across airways. Airways are tubular structures and their geometry is determined by the centreline given by bronchi lumen centre. These centrelines have a tree structure given by bronchi branching levels. Then, to quantify the anatomical consistency with segmentations, we analyse the geometry of their skeleton. To do so, the segmentation skeleton is encoded in a graph that represents its branching geometry by nodes and edges. The nodes of the graph correspond to the skeleton branching points and its edges represent branch connectivity. The trachea entry point allows directing the graph using the depth first search algorithm. The directed graph is a (binary) tree, where levels correspond to bronchial levels and leafs correspond to the most distal points achieved by the segmentation.

The directed graph of the final segmentation skeleton is encoded using two adjacency matrices: one binary matrix defining node tree connectivity and one matrix of 3D segments that keeps the list of 3D skeleton points that connect each pair of adjacent nodes. This scheme is flexible and allows us to develop a matching between a CT-video bronchial structure and the coded airways using anatomical landmarks [21].

### 3.3 Path encoding

Each level of the binary tree represents a bronchial depth *d*. A navigation path is defined as a sequence of segments $S_1\comma \; \, S_2\comma \; \, \ldots \comma \; \, S_n$, connecting a leaf node in $S_n$ to the trachea (root node). Each segment $S_d$ contains the sequence of skeleton points $p_1^d \comma \; \, p_2^d \comma \; \, \ldots \comma \; \, p_{kd}^d $ that joins two bifurcation points at consecutive levels $d - 1$ and *d*, being $p_{kd}^d $ the bronchial bifurcation point at level *d*.

**Fig. 3:**
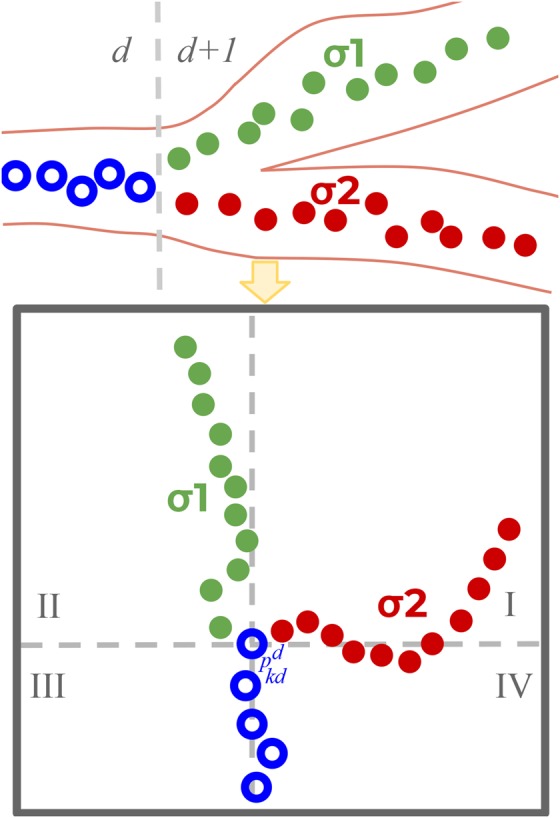
Codification of a path, using the 2D position of airways segments in the projection

For encoding the path, each segment is labelled according to its 2D position in the projected images. These positions are obtained by the simulation of virtual guiding using CT scans of patients. As illustrated in Fig. 3, for each bifurcation point $p_{kd}^d $ along a path, a virtual camera is located with the viewing direction to its children segments, specifically $\sigma _1$ and $\sigma _2$. The skeleton points of $\sigma _1$, $\sigma _2$ are projected from that point of view to label $\sigma _1$, $\sigma _2$ according to its position in the projected image.

Each projected image is split into four quadrants (labelled I, II, III, and IV) centred at the projection of $p_{kd}^d $. Each projected point belongs to a quadrant and $\sigma _1$, $\sigma _2$ are assigned to a quadrant to which their points belong to most frequently.

Fig. 3 illustrates the segment quadrant assignment. The grey-dotted lines show the quadrant division and $\sigma _1$, $\sigma _2$ points are a plot in different colours. We observe that $\sigma _1$ has points in the quadrant I and IV, but it is assigned to quadrant I which is where the majority of $\sigma _1$ points belong.

### 3.4 Navigation instructions

The classification of each segment allows constructing a set of ordered instructions using the quadrants of the segments, connecting the trachea to a peripheral lesion. The based-quadrant instructions are described by their position in images: I is named as *up-right*, II as *up-left*, III as *down-left*, and IV as *down-right*. The labels of segments given by their quadrant are further simplified: the common word in the projected segments at each bifurcation is excluded. For instance, if two segments are in quadrants I/II and III/IV, the instruction whether is *go up* or *go down*, respectively; if they are in quadrants I/IV and II/III, the instruction whether is *go right* or *go left*. If two segments are in the same quadrant, we order them by their angle inside the quadrant they belong, after that the same approach to get a single instruction is applied.

**Fig. 4:**
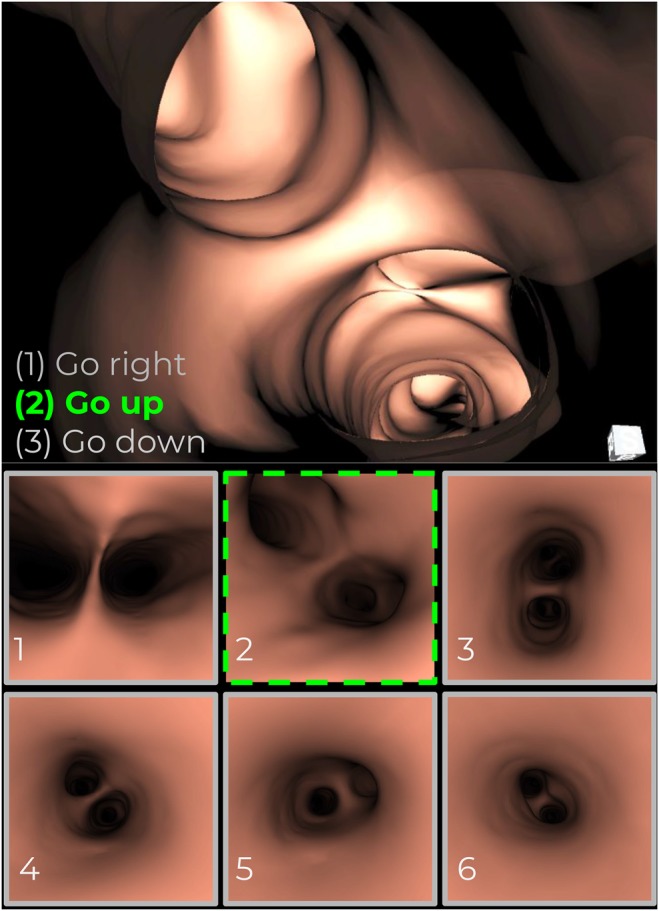
Instructions presented to physicians as a mosaic of images

The instructions are visually presented to physicians as a mosaic of images representing each bifurcation point. Consequently, physicians can navigate through each one using the provided GUI of BronchoX, as shown in Fig. 4. Each bifurcation is enumerated according to its bronchial level, presenting the navigation instructions in a simple way.

## 4 Results and discussion

BronchoX is developed using open-source available software packages, also the user interface is built using QT forms, and the 3D airway model is an OBJ geometrical file. About the 3D model, this is visualised with 70% transparency using the dual depth peeling algorithm [22], with an occlusion ratio = 0.1, and a maximum of 40 layers. The code is available as open source at https://gitlab.com/esmitt/BronchoX, where the latest version can be found and where all developments can be followed.

The segmentation was applied to the 40 CT scans data of the Medical Image Computing & Computer Assisted Intervention Conference Challenge EXACT'09 [23], acquired in different conditions including variable slice thickness (0.5–1.0 mm), in-plane voxel sizes (0.55–0.78 mm), and radiation dose (120/140 kVp, 10.0–411.5 mAs). Evaluation of EXACT'09 cases show that our method achieves competitive performance.

From a technical point of view, it is a self-contained software. It could execute scripts written in MATLAB, which are used to perform the segmentation process. Also, the VTK, ITK, and QT libraries are integrated into the same code, offering a CMake configuration to be compiled in different configurations (i.e. different compilers and operating system).

To validate the reliability of our proposal, physicians verified the effectiveness of a set of given instructions to reach distal bronchioles. For this, we generated different virtual explorations using CT volumes of ten anonymised patients. CT scans were acquired with a 320-detector row Aquilion ONE, Toshiba CT scan, and a sample thickness of 0.5 mm. For each patient, four virtual explorations covering four lobes were defined: the left and right upper lobes (LUL and RUL) and left and right lower lobes (LLL and RLL). For each path, a sequence of instructions was validated by three experts, trying to reproduce the path using the given instructions. The generated paths are between the 6th and 12th bronchial levels.

We defined a false instruction rate (FIR) variable, representing when an expert could not reproduce the given path. This data is modelled as a mixed model using R, version 3.2.5. A Poisson model was adjusted to include the segmental lobe as a factor (Lobe), and a random subject effect (Pat) to consider the intra-individual variability among cases, and a random effect to model inter-observer variability as

$$\log \lpar {\rm FI}{\rm R}_{ijk}\rpar = \beta _0 + \beta _1{\rm Lobe} + {\rm Pa}{\rm t}_i + {\rm Ob}{\rm s}_j + \varepsilon _{ijk}$$

where ${\rm Pa}{\rm t}_i{\rm \sim }N\lpar 0\comma \; \, \sigma _{{\rm Pat}}\rpar $ denotes the random effect that models intra-patient variability, ${\rm Ob}{\rm s}_j{\rm \sim }N\lpar 0\comma \; \, \sigma _{{\rm Obs}}\rpar $ the random effect of inter-observer variability, and the factor Lobe with values RLL, LLL, RUL, and LUL. Model assumptions were validated by means of residual analysis and influential values. Also, model coefficients, *p* values, and 95% confidence interval (CI) for significance in the main effects were computed. The CIs were back transformed to the original scale for their interpretation. Thus, a *p* value of <0.05 was considered statistically significant.

Descriptive statistics [average and standard deviation (SD)] and model adjustment for FIR are shown in Table 1, in a percentage way. The increase in FIR for the lower-left lung lobe is mostly due for confusing instructions at the third generation, just after the LUL–LLL branching point. Although the 3D geometry around the third generation presents two branching points (i.e. two levels), they are not appreciated in the projected images due to a short distance between them.

**Table 1:** FIR values for the four lobes (descriptive and model)

% FIR	Descriptive		Model		
	Mean	SD	Coeff	*p*-val	CI
RLL	2.8	4.6	1	—	(0.6, 5.2)
LLL	13	11.7	1.3	<0.01	(5.2, 20.6)
RUL	1.7	4.0	−0.35	0.06	(0.1, 3.4)
LUL	6.4	8.7	0.64	0.08	(2.1, 10.5)

**Fig. 5:**
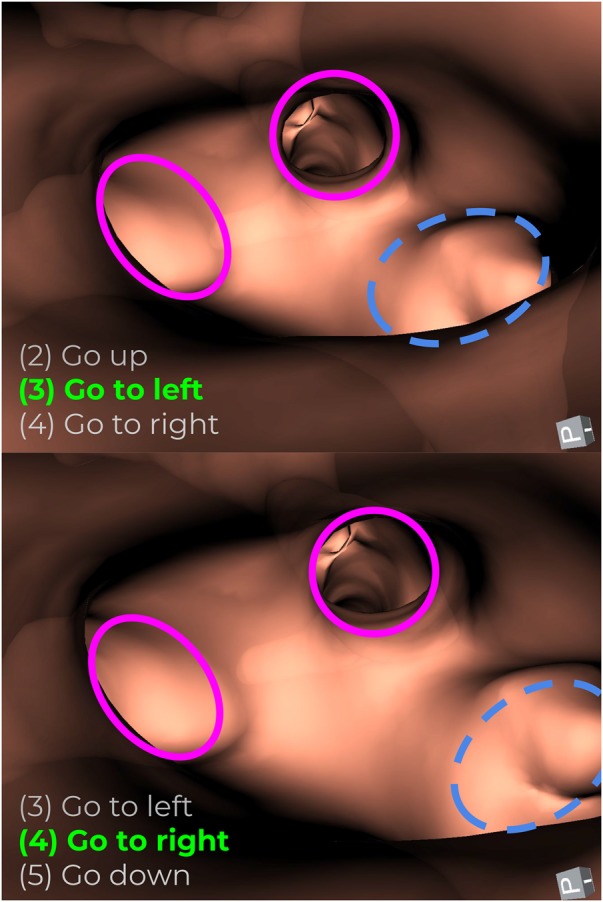
Example of the phenomenon, when three lumens appear in a projection (third bronchial level)

In projected images, the LLL lumen is hard to identify visually, then three airway lumens that correspond to the projection of LLL next generation are visible. Therefore, from the point of view of the operator (i.e. bronchoscopist), there are three possible airways to follow in the same level, whereas in our codification consist of two consecutive levels with two airways each one. Fig. 5 illustrates this phenomenon, where three lumens appear in the projection for a particular bronchial level.

The upper part of Fig. 5 corresponds with a captured view at depth level $d = 3$, and the lower part when $d = 4$ for one example path. In the same projection, there are both a child from level 3 (blue – dotted ellipse) and children of level 4 (purple – continuous ellipse). This might generate confusing instructions to be followed by operators. This occurrence happens at the short distance between a bifurcation point and the segment's children of the next level.

## 5 Conclusions

An exploration bronchoscopy software for biopsy intervention planning and navigation is introduced in this Letter. The developed VB software, named BronchoX, has an architecture designed to create personalised applications; these applications could be implemented as supporting software in the planning stage and subsequently on the surgery stage. Similarly, it is possible to add new features over the C++/QT layer to improve the solution. For example, the addition of the Pulse Physiology Engine (https://physiology.kitware.com.) might simulate the comprehensive patient physiology in the respiratory system. Besides, the integration of external devices or libraries as the Intel Real Sense (https://realsense.intel.com/.) or any augmented reality/virtual reality library is possible.

BronchoX has many interesting functionalities for VB navigation. It is possible to identify a lesion using the anatomical planes and to compute the path to a lesion's closest point. The path is encoded as a sequence of instructions at each airway bifurcation to traverse. Instructions are given in a natural language, allowing the easy identification of the bronchi that physicians should follow. This feature offers a great advantage in order to follow the planned roadmap.

The results based on mixed models are very promising, they provide a quantitative analysis of performance considering both population and anatomical factors. The statistical analysis gives a bias in instructions for the left lower lobe, being introduced by the close spatially consecutive levels. As a result, these levels are visualised as a single level with three lumens, when certainly is composed by two levels. Then, unifying those levels into one single instruction instead of two seems like a practical solution.

Future work will concentrate on introducing a hands-free guiding system to be used in the operating room, to guide during *in vivo* explorations. This might be realised with a specific hardware, also it will be integrated as part of our layer-based architecture. Besides, we want to integrate the video-bronchoscopy landmarks [24] into BronchoX to obtain a synchronisation between the virtual and *in vivo* video patient traversal.

We have confidence that our work might be a starting point for supporting researchers into the development of new algorithms using our open-source solution. BronchoX is the first step in the development of a bronchoscopy navigation and exploration system for physicians, and it will be as a foundation to develop further studies in VB.

## 6 Funding and declaration of interests

The authors thank NVIDIA for the Titan X Pascal used for this research. This work was supported by Catalan, Spanish and European projects DPI2015-65286-R, 2014-SGR-1470, CERCA Programme/Generalitat de Catalunya. Esmitt Ramírez holds the fellowship number BES-2016-078042 granted by the Ministry of Economy, Industry and Competitiveness, Spain. Carles Sánchez is supported by the ACCIO Tecniospring TECSPR17-1-0045 Program. Debora Gil holds a Serra Húnter Fellow.

## 7 Conflict of interest

None declared.
